# Effects of NMDA receptor antagonists on working memory and gamma oscillations, and the mediating role of the GluN2D subunit

**DOI:** 10.1038/s41386-025-02129-9

**Published:** 2025-05-15

**Authors:** Chitra Vinnakota, Matthew R. Hudson, Kazutaka Ikeda, Soichiro Ide, Masayoshi Mishina, Suresh Sundram, Nigel C. Jones, Rachel Anne Hill

**Affiliations:** 1 Department of Psychiatry, Monash University, Clayton, VIC 3168 Australia; 2 Department of Neuroscience, Monash University, Melbourne, VIC Australia; 3 Addictive Substance Project, Tokyo Metropolitan Institute of Medical Science, Tokyo, 156-8506 Japan; 4 Department of Neuropsychopharmacology, National Center of Neurology and Psychiatry, Tokyo, 187-8553 Japan; 5 Brain Science Laboratory, The Research Organization of Science and Technology, Ritsumeikan University, 1-1-1 Nojihigashi, Kusatsu, Shiga Japan; 6 Mental Health Program, Monash Health, Clayton, VIC 3168 Australia; 7 Department of Neurology, The Alfred Hospital, Commercial Road, Melbourne, VIC 3004 Australia

**Keywords:** Working memory, Cellular neuroscience

## Abstract

Working memory relies on synchronised network oscillations involving complex interplay between pyramidal cells and GABAergic interneurons. NMDA receptor (NMDAR) antagonists influence both network oscillations and working memory, but the relationship between these two consequences has not been elucidated. This study aimed to determine the effect of NMDAR antagonists on network oscillations during a working memory task in mice, and the contribution of the GluN2D receptor subunit. After training wildtype (WT) and GluN2D-knockout (KO) mice on the Trial-Unique-Non-match to Location (TUNL) touchscreen task of working memory, recording electrodes were implanted into the prefrontal cortex (PFC) and hippocampus. Mice were challenged with either (S)-ketamine (30 mg/kg), (R)-ketamine (30 mg/kg), phencyclidine (PCP, 1 mg/kg), MK-801 (0.3 mg/kg) or saline prior to TUNL testing while simultaneous local field potential recordings were acquired. PCP disrupted working memory accuracy in WT (*p* = 0.001) but not GluN2D-KO mice (*p* = 0.79). MK-801 (p < 0.0001), (S)-ketamine (p < 0.0001) and (R)-ketamine (*p* = 0.007) disrupted working memory accuracy in both genotypes. PCP increased baseline hippocampal gamma (30–80 Hz) power in WT (*p* = 0.0015) but not GluN2D-KO mice (*p* = 0.92). All drugs increased baseline gamma power in the PFC in both genotypes (p < 0.05). Low gamma was induced during the maintenance phase of the TUNL task and increased when mice correctly completed the task (*p* = 0.024). This response-dependent increase in low gamma was disrupted by all drugs. In summary, PCP action involves the GluN2D subunit of the NMDA receptor in the hippocampus to alter baseline gamma power and working memory. Task-induced low gamma activity during maintenance aligns with task performance, and is disrupted by all NMDAR antagonists.

## Introduction

Schizophrenia affects 0.29–0.7% [1, 2] of the world’s population but causes significant social and economic burden [2]. Approximately 80% of individuals with schizophrenia are unemployed and it is one of the top 20 causes of years lived with disability globally [3]. The economic impact of schizophrenia is estimated between 0.30–0.60% of GDP in high income countries incorporating both direct medical treatment costs as well as indirect economic loss related to criminal justice/homelessness, loss of tax revenue, and productivity losses for both people with schizophrenia and their caregivers [4, 5]. While schizophrenia is characterised by positive symptoms that “add” to one’s psyche, including delusions and hallucinations, as well as negative symptoms that “take away” from one’s psyche, such as social withdrawal, anhedonia and disordered thinking, cognitive symptoms are the best predictor of functional outcomes such as employment, and independent living [6]—outcomes that carry significant financial burden. Cognitive symptoms include impairments in working memory, verbal learning, visual learning, attention, reasoning and problem solving and social cognition [7]. However, working memory (WM) in particular has been specifically related to long-term community functioning and as such has been identified as a core feature of schizophrenia [8, 9]. Importantly, WM deficits are not treated by current antipsychotic medications, therefore there is an urgent need to better understand the mechanisms causing WM impairments in schizophrenia in order to develop evidence-based treatments.

Neural oscillations play a crucial role in facilitating information processing and communication within and across brain regions [10,11]. Gamma oscillations (30–100 Hz), in particular, are implicated in higher-order cognitive and sensory processing [12, 13] and are evoked when humans perform tasks that require WM, attention, emotional processing and perception [14–18]. In people with schizophrenia increased baseline (or ongoing) gamma power but reduced cognitive task-induced gamma power, compared with healthy humans has been reported [19–23]. These aberrant changes in both resting-state and induced gamma oscillations have been linked to cognitive symptoms in people with schizophrenia [20, 24]. For example, one study reported a significant negative correlation between resting-state gamma power and performance of a verbal learning task in people with schizophrenia [20], while another reported significantly reduced gamma oscillations during a WM task in people with schizophrenia compared with controls [24]. Understanding neural oscillation dysfunction in schizophrenia may provide clues as to how to better treat WM impairment.

NMDAR antagonists are well known to recapitulate the full spectrum of behavioural symptoms relevant to schizophrenia, and have therefore been used as tools to model schizophrenia symptoms in rodents [25]. Interestingly, NMDAR antagonists evoke similar gamma oscillatory changes to those found in schizophrenia. NMDAR antagonists like ketamine, PCP and MK-801 have repeatedly been shown to increase ongoing gamma power in the cortex and hippocampus and decrease stimulus-evoked gamma power in humans and rodent models [26–33]. The oscillatory and behavioural effects of these drugs promote the acute NMDAR antagonist model as one which can be used to explore the interrelationships between behaviour and electrophysiology relating to schizophrenia [34]. However, there are subtle differences in binding affinity and potency of various NMDAR antagonist drugs. For example, ketamine has a much shorter half-life than PCP or MK-801, and MK-801 shows higher affinity for NMDA receptors containing GluN2A/B subunits while PCP shows greater affinity for GluN2B-D subunits [35]. Different potencies of the two ketamine enantiomers, (S)-ketamine and (R)-ketamine are also a major consideration, particularly when considering the current literature surrounding their antidepressant efficacy. (R)-ketamine is a more potent NMDAR antagonist and studies have shown that its antidepressant effects are greater and longer lasting, with fewer side effects [36]. Studying the differential impact of these drugs on neural oscillations during working memory allows us to uncover the most appropriate model for testing working memory dysfunction associated with schizophrenia and also provides further information on which NMDAR subunits are likely key contributors to working memory.

Although the precise biophysical and cellular mechanisms underlying neuronal oscillatory activity are not clear, there is extensive evidence implicating fast-spiking PV interneurons in the generation of neural oscillations within the gamma frequency range [37–41]. PV interneurons receive NMDAR-mediated excitatory input from pyramidal cells and in turn modulate neural network activity via synchronous and co-ordinated GABAergic inhibition of local excitatory neurons, resulting in gamma oscillations [10, 39, 42]. One leading theory for the aberrant gamma oscillatory changes in schizophrenia is the hypofunction of NMDARs on PV interneurons [43–45]. GluN2D-containing NMDARs are especially enriched in PV interneurons [46, 47], and reductions in PCP and ketamine-induced hyperlocomotion in GluN2D-knockout (KO) mice [48, 49] as well as reduced ketamine-induced increase in baseline gamma power in GluN2D-knockout (KO) mice compared with WT mice [50], have been previously reported. Here, the objective of this study was to explore NMDAR antagonist-induced changes in neuronal oscillations in GluN2D-KO compared with WT mice during the performance of a WM task.

There are several different methods to assess cognition in animal models. However, many of these methods bear little resemblance to how cognition is assessed in humans, which may contribute to the current lack of effective therapies for cognitive symptoms in schizophrenia. The Cognitive Neuroscience Treatment Research to Improve Cognition in Schizophrenia (CNTRICS) initiative was established in response to this need for therapies that improve functional outcomes in patients [51]. One of the main goals of CNTRICS was the development of tasks with a high degree of cognitive construct validity which could be used both in humans and in animal models. The rodent operant touchscreen system was thus created with the aim of closely resembling assessments of cognition used clinically to improve translatability and maximise efficiency in identifying appropriate therapeutic targets and treatments for schizophrenia [52]. The touchscreen system is translational, automated, non-aversive, low-stress, able to assess multiple cognitive domains within the same testing environment and can detect both impairments and enhancements in function [52, 53]. This study utilises the trial-unique non-match to location (TUNL) touchscreen-based task to assess the influence of NMDAR antagonists on working memory in wild type (WT) and GluN2D-KO mice. In addition, for the first time, we combine touchscreen testing with simultaneous measurement of electrophysiological signals during task performance with the goal of identifying neural oscillation patterns which underpin drug and genotype effects on WM.

## Materials and methods

### Animals and housing

GluN2D-KO mice were obtained from the Tokyo Metropolitan Institute of Medical Science and transported to the Monash Animal Research Platform, Monash Medical Centre (Clayton, Victoria, Australia) where a breeding colony was established. GluN2D heterozygous mice were bred to obtain WT, heterozygous and homozygous GluN2D-KO male and female littermates. At 6–7 weeks of age, mice were transferred from the breeding facility to the laboratories in the Department of Neuroscience, School of Translational Medicine, Monash University (Prahran, Victoria, Australia) where all husbandry, housing, and behavioural testing was undertaken. All mice (*n* = 42) were housed in groups of 2–5 in individually ventilated cages (Techniplast, NSW, Australia) with a reversed 12-h dark-light cycle (lights off at 9:30 am) allowing experiments to be conducted during the active phase of the mouse circadian cycle. Cages were monitored daily and changed weekly. After allowing mice to acclimatize to reverse light conditions (2 weeks), a food-restricted diet was gradually introduced (with water available *ad libitum*), until mice reached 85–90% of their initial free-feeding weight, which was maintained throughout the testing period. All procedures were approved by the Monash University Animal Ethics Committee (Project #: E/1837/2018/M).

### Trial Unique Non-Matching to Location (TUNL) task of working memory

The TUNL task was performed in the automated touchscreen operant chambers (Campden Instruments Ltd., UK) for mice, and Whisker and Abet II software (Campden Instruments Ltd., UK) were used to control the system and for data collection as previously described [54, 55].

The TUNL task was conducted as previously described [53–56] (see supplementary Table 1 for TUNL training protocol). Each TUNL session consisted of a maximum of 48 trials, each comprising two phases: during the sample phase, an initiation triggered by an IR beam break near the reward collection tray would result in the illumination of one window out of five possible locations. After the mouse nose-poked this stimulus, the stimulus disappears and a varying delay period begins. A second initiation triggers the start of the subsequent (choice) phase where a new location appears alongside the sample location requiring the mice to recall the sample location and choose the new location for a food reward. If the mouse chooses correctly, the delivery of the food reward is followed by an inter-trial interval (ITI) before the next trial. Whereas, a nose-poke to the incorrect location leads to a 5-s timeout and the initiation of a correction trial, where the same stimuli are presented repeatedly until a correct response is achieved. One session was conducted per mouse per day, occurring six days a week except for the pharmacological studies during which there was a minimum gap of 48 h between consecutive treatments. During probe sessions mice were tested using S1c trials with a delay of 1 s between the sample and choice phase as our previous findings indicated that mice tend to use working memory rather than other strategies, like side bias, when tested using this configuration [54].

### Drug challenge

Mice were injected with either vehicle (0.9% saline) or S-ketamine (S-ket) (3, 10, and 30 mg/kg), R-ketamine (R-ket) (3, 10 and 30 mg/kg), PCP (1 and 3 mg/kg) or MK-801 (0.1 and 0.3 mg/kg) as previously described [48]. All mice randomly received each dose of all treatments with at least 48 h in between treatments. Each compound was delivered via a single intraperitoneal (i.p.) injection at 10 µl/g. Drugs were administered 10 mins before the testing session began for S-ket, R-ket and PCP and 30 mins before the testing session for MK-801. We waited 30 min before testing with MK-801 as the drug causes mice to be quite hyperactive so it was necessary to wait until this dampened before placing them into the chambers. The primary outcome measure was task accuracy. During the drug challenge, the experimenter was blinded to the genotype of the mice but not the drug type or dose.

### Electrophysiological procedures

Following the completion of behavioural experiments, mice underwent surgery to implant recording electrodes in the medial prefrontal cortex (mPFC) and dorsal hippocampus (dHPC) as previously described [57, 58], see also supplementary methods. We have previously validated the electrode placement coordinates [32].

Once TUNL performance was re-established post-surgery with the head-stage cable attached, mice were exposed to vehicle (0.9% saline), S-ket (30 mg/kg), R-ket (30 mg/kg), PCP (1 mg/kg) or MK-801 (0.3 mg/kg) via a single i.p. injection at 10 µl/g and were tested in TUNL probe sessions where combined electrophysiological and TUNL behavioural data were simultaneously recorded. Whisker and Abet II software (Campden Instruments Ltd., UK) were used to collect behavioural data whilst the electrophysiological data was acquired using Multi Channel Systems software (Harvard Biosciences Inc., USA). A modified ABET TUNL schedule was used which allowed behavioural data to be synchronised with electrophysiological data (see supplementary methods).

### Electrophysiological analysis

The time-stamped electrophysiology data was imported from Neuroexplorer (Plexon, USA) and analysed using custom-designed MATLAB (MathWorks, USA) scripts (see supplementary methods). Baseline activity oscillatory power was first assessed from a 5 s window within the 12 s intertrial interval (ITI) preceding each trial (see supplementary methods). Within the ITI mice are not actively engaged in the task. TUNL task-related oscillatory activity was then analysed. Here, continuous LFP data was segmented into epochs from 0 to 2000 ms prior to selecting the sample stimulus (i.e.: the encoding phase), 0–2000 ms immediately following selection of the sample stimulus (i.e.: maintenance phase) and 0–2000 ms prior to selection of the choice stimulus (i.e.: retrieval phase) - see Fig. 4A. Epochs were also categorised according to whether the mouse made a correct or incorrect choice in that specific trial (see supplementary methods). Change in power was calculated as the average power during each of the phases (encoding, maintenance or retrieval) / baseline average power for each animal in each frequency band. Low gamma was taken from between 30–40 Hz while gamma power is between 30–100 Hz.

### Statistical analysis

All statistical analyses and graphical representations were generated using GraphPad Prism (version 8.3.1, GraphPad Software, San Diego). For the behavioural and baseline electrophysiological data, a repeated measures three-way ANOVA was performed with genotype, sex and treatment as between group factors. Sphericity was assumed for each test. There was no main effect of sex or interaction of drug or genotype with sex, therefore sexes were consolidated. Data were then assessed by either a repeated measures 2-way ANOVA or mixed model depending on whether there were missing data points.

For the task-evoked oscillatory changes, a repeated measures two-way ANOVA was performed with response and time as between factors to determine change in power according to whether mice chose the correct or incorrect response, and over the time course of the task. Sphericity was assumed for each test. Two-way ANOVAs were then performed to assess the impact of drug and genotype on average low gamma across all trials. To assess change in low gamma in correct versus incorrect trials, paired t tests for each group were performed. Outliers were removed by means of the ROUT test (Q = 5%). In all cases, the significance level was set to p ≤ 0.05. Power analysis for 80% power using the 3-way ANOVA design requires *n* = 8 for a medium effect (0.75).

## Results

### PCP does not disrupt working memory in GluN2D-KO mice in contrast to other NMDAR antagonists

**Fig. 1:**
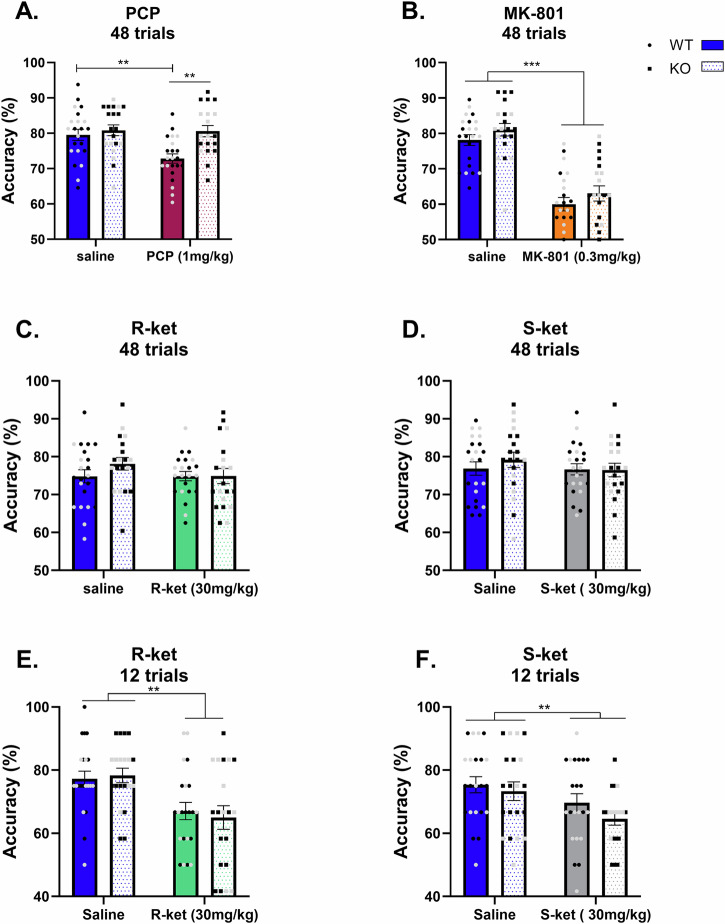
The effects of NMDAR antagonists on working memory in WT and GluN2D KO mice. Working memory accuracy in WT (solid bars) and GluN2D KO (patterned bars) mice administered PCP (**A**), MK-801 (**B**), R-ket (**C**) or S-ket (**D**) over 48 trials. Working memory accuracy over the first 12 trials following R-ket (**E**) and S-ket (**F**) administration. All drugs impair working memory accuracy, however, for PCP this was only observed in WT mice, not KO mice. Also, for R-ket and S-ket, disruptions to working memory are only observed during the first 12 trials. *n* = 12 WTM, 10 WTF, 12 KOM, 8 KOF. Data are mean ± SEM. **p < 0.01, ***p < 0.0001. Black dots are male mice and grey are females.

Mice were trained until they reached a criterion of 80% accuracy on the TUNL task over 3 consecutive days. Once they reached this criterion they were then challenged 10 min prior to the task with either saline, PCP, R-ket or S-ket and 30 min prior to the task with MK-801. Supplementary Table 2 shows the working memory accuracy across all groups and sexes and multiple drug concentrations. There was no main effect of sex or interaction of sex with genotype or drug effects, therefore male and female data was consolidated for further statistical comparisons. Figure 1 shows working memory accuracy following administration of one selected dose of either PCP, MK-801, R-ket or S-ket with the sexes consolidated due to lack of significant interactions of sex. Full statistical outputs for working memory accuracy are in Supplementary Table 3. d.

Figure 1A shows a significant effect of PCP (F (1, 79) = 5.395, *p* = 0.023), a significant effect of genotype (F (1, 79) = 9.126, *p* = 0.0034) and a significant PCP x genotype interaction (F (1, 79) = 4.673, *p* = 0.034) on working memory accuracy. Here, Šídák’s multiple comparisons test showed a significant difference between WT and KO mice only in the PCP treated group (*p* = 0.001), not in saline treated mice (*p* = 0.79) and PCP disrupted working memory accuracy in WT (*p* = 0.003) but not GluN2D-KO mice compared to saline (*p* = 0.99) (Fig. 1A). MK-801 disrupted working memory in both genotypes as seen by a main effect of MK-801 (F (1, 40) = 87.16, p < 0.0001) but no effect of genotype and no genotype x drug interaction (Fig. 1B). For R-ket and S-ket (Fig. 1C, D) there was no effect of drug, genotype or interaction when assessing all 48 trials of the TUNL task. However, when assessing the first 12 trials (approximately first 15 min of the test) we found a main effect of drug for both R-ket (F (1, 40) = 15.32, *p* = 0.0003) and S-ket (F (1, 40) = 7.235, *p* = 0.010). However, there was no effect of genotype and no genotype x drug interaction. Therefore, all NMDAR antagonist drugs disrupt working memory, however, for PCP this is only true in WT, not GluN2D-KO mice.

### PCP does not increase baseline hippocampal gamma power in GluN2D-KO mice in contrast to other NMDAR antagonists

**Fig. 2:**
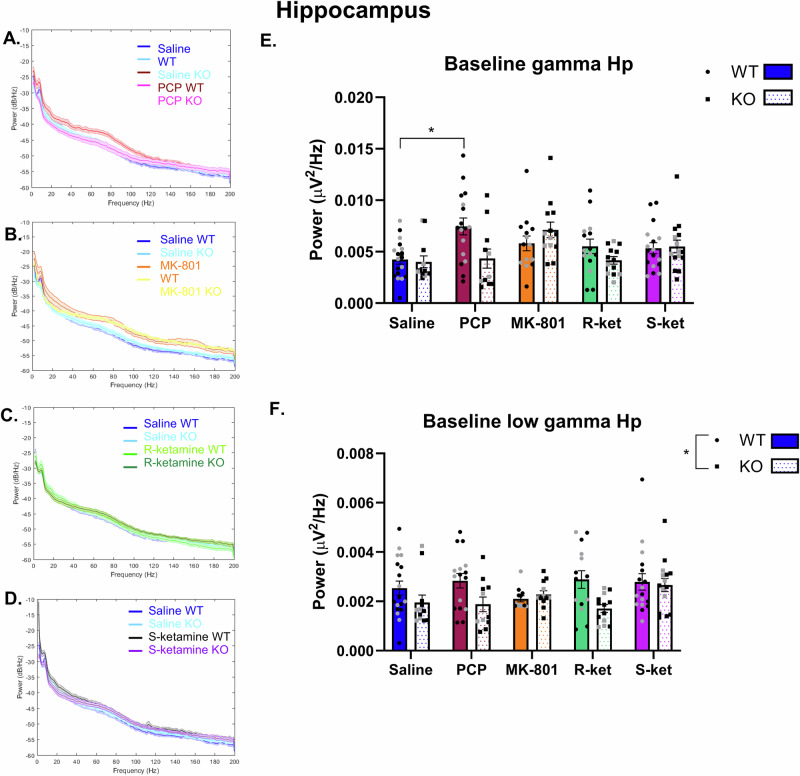
The effects of NMDAR antagonists on baseline hippocampal gamma and low gamma power in WT and GluN2D KO mice. Baseline power spectral density depicting power on the abscissa and frequency on the ordinate in the hippocampus of WT and KO mice treated with either saline, PCP (**A**), MK801 (**B**), R-ket (**C**) or S-ket (**D**). **E** PCP increased hippocampal gamma power in WT but not GluN2D-KO mice, while GluN2D-KO mice show decreased hippocampal low gamma power (**F**). MK-801, R-ket and S-ket had no significant effect of hippocampal gamma (**E**) or low gamma power (**F**) at baseline. *n* = 18 WT saline (10 M/8 F), 12 KO saline (7 M/5 F), 17 WT PCP (11 M/6 F), 11 KO PCP (8 M/3 F), 14 WT MK-801 (8 M/6 F), 13 KO MK-801 (9 M/4 F), 15 WT R-ket (9 M/6 F), 13 KO R-ket (8 M/5 F), 17 WT S-ket (9 M/8 F) and 15 KO S-ket (10 M/5 F). Data are Mean ± SEM. *p < 0.05. Black dots are male mice and grey are females.

Baseline gamma power was calculated as the average power recorded across all intertrial intervals within the TUNL task—a period whereby mice were in the touchscreen chambers but not actively performing the task. Power frequency plots were generated for each drug challenge (Fig. 2A–D). To assess and compare the effects of each drug on baseline gamma power in both WT and GluN2D KO mice a mixed-effects model was applied with drug and genotype as the fixed effects. Full statistical outputs for baseline hippocampal gamma and low gamma are in Supplementary Table 4. In the hippocampus, there was a main effect of drug (F (3.309, 81.90) = 5.012, *p* = 0.002) and a drug x genotype interaction (F (4, 99) = 3.336, *p* = 0.013, Fig. 2E). Šídák’s multiple comparisons test showed a significant increase in baseline gamma power in WT PCP vs WT saline treated mice (*p* = 0.046) but no such effect of PCP in KO mice (*p* = 0.99). However, the difference between WT and KO PCP treated groups only approached significance (*p* = 0.09). For the main effect of drug there was only a significant effect of PCP compared to saline (*p* = 0.049), while MK-801 (*p* = 0.117), R-ket (*p* = 0.99) and S-ket (*p* = 0.26) had no significant effect on baseline gamma power. For hippocampal low gamma, there was no effect of drug, but there was a significant effect of genotype (F (1, 36) = 7.138, *p* = 0.011), with GluN2D KO mice showing reduced baseline low gamma (Fig. 2F).

**Fig. 3:**
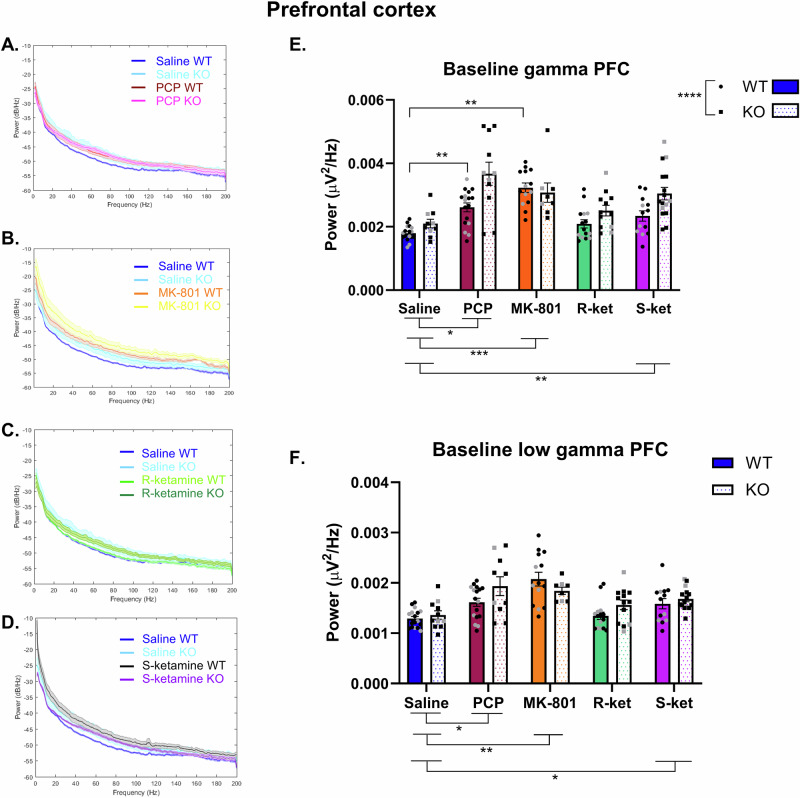
The effects of NMDAR antagonists on baseline PFC gamma and low gamma power in WT and GluN2D KO mice. Power spectral density plots of PCP (**A**), MK801 (**B**), R-ket (**C**) and S-ket (**D**) occurring in hippocampus of WT and GluN2D-KO mice. PCP and MK-801 significantly increased baseline gamma in WT but not KO mice, while S-ket increased baseline gamma power in both WT and KO mice (**E**). KO mice show increased baseline gamma power. PCP, MK-801 and S-ket all increased baseline low gamma in the PFC with no effect or interaction with genotype (**F**) *n* = 17 WT saline (10 M/7 F), 11 KO saline (8 M/3 F), 17 WT PCP (11 M/6 F), 11 KO PCP (8 M/3 F), 14 WT MK-801 (10 M/4 F), 8 KO MK-801 (6 M/2 F), 15 WT R-ket (9 M/6 F), 12 KO R-ket (9 M/3 F), 13 WT S-ket (9 M/4 F), 16 KO S-ket (11 M/5 F). Data are Mean ± SEM. *p < 0.05, **p < 0.01, ***p < 0.0001. Black dots are male mice and grey are females.

Full statistical outputs for baseline PFC gamma and low gamma are in Supplementary table 4. Power frequency plots were generated for each drug challenge (Fig. 3A–D).

In the PFC, there was a significant effect of drug (F (2.752, 84.62) = 15.94, p < 0.0001) a significant effect of genotype (F (1, 123) = 15.91, *p* = 0.0001) and a significant drug x genotype interaction (F (4, 123) = 2.847, *p* = 0.026) for baseline gamma power (Fig. 3E). The significant effect of genotype showed that KO mice had higher baseline gamma power across the groups. For the main effect of drug, Šídák’s multiple comparisons test showed a significant difference between saline vs. PCP (*p* = 0.018), saline vs. MK-801 (*p* = 0.0002) and saline vs. S-ket (*p* = 0.001) with all drugs increasing gamma power. However, there was no significant difference between saline and R-ket (*p* = 0.208). When exploring post-hoc findings from the genotype x drug interaction, while there was no significant effect of genotype within each of the drug groups (see Supplementary table 4), WT but not KO mice showed a significant increase in gamma power when treated with PCP (WT saline vs. PCP, *p* = 0.005) or MK-801 (WT saline vs. MK-801, *p* = 0.002).

For baseline low gamma power there was only a main effect of drug (F (2.884, 62.73) = 14.03, p < 0.0001) and no significant effect of genotype or genotype x drug interaction (see Supplementary Table 4). Multiple comparisons test for the main effect of drug indicated significant effects of PCP (*p* = 0.02), MK-801 (*p* = 0.002) and S-ket (*p* = 0.01) on low gamma with all drugs increasing power. R-ket however, had no significant effect on low gamma power (*p* = 0.75).

### Task-induced change in low gamma power increased during maintenance when mice chose the correct response

**Fig. 4:**
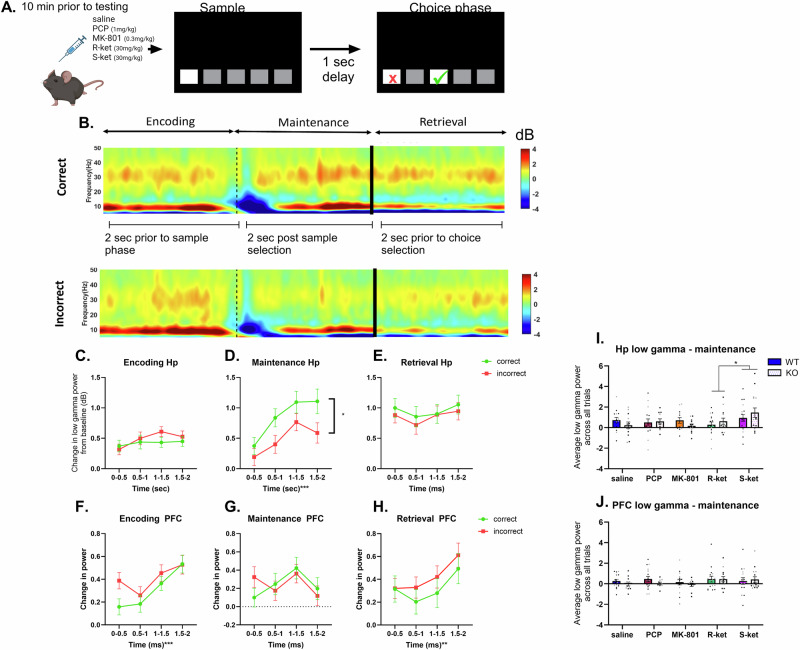
The effects of NMDAR antagonists on working memory task-induced gamma power in WT and GluN2D KO mice. **A** Schematic illustration depicting the inter-trial interval from which baseline recordings are captured and the sample and choice phases of the TUNL task. **B** Hippocampal spectral power heat map of all trials from 5 to 50 Hz in all three phases of the TUNL task relative to baseline periods; The top panel represent data from correctly answered trials, whereas the bottom panel represents data from incorrect trials. The thin dotted line represents the time at which a mouse completes the encoding phase and initiates the maintenance phase. The thick black line represents a variable duration of time, reflective of variable maintenance phase duration across trials. **C** During the encoding phase hippocampal low gamma is unchanged by time or by response type. **D** During the maintenance phase hippocampal low gamma increases as mice are about to select the choice phase selection and in addition low gamma power is increased when mice go on to make the correct choice. **E** During the retrieval hippocampal phase low gamma is unchanged by time or by correct or incorrect response. **F** During the encoding phase PFC low gamma is changed by time but not  by response type. **G** During the maintenance phase PFC low gamma is unchanged by time or by response type. **H** During the retrieval phase PFC low gamma is changed by time but not  by response type. **I** Change in hippocampal low gamma during the maintenance phase is significantly different between, S-ket and R-ket treatment groups, but is unaffected by genotype or drug in the PFC (**J**) *n* = 16 WT saline (9 M/7 F), 13 WT PCP (7 M/6 F), 12 WT MK-801 (6 M/6 F), 12 WT R-ket (7 M/5 F), 17 WT S-ket (9 M/8 F), 13 KO saline (8 M/5 F), 11 KO PCP (9 M/2 F), 9 KO MK-801 (6 M/3 F), 12 KO R-ket (8 M/4 F), 12 KO S-ket (8 M/4 F). Data are Mean ± SEM. *p < 0.05. Black dots are male mice and grey are females.

We next sought to determine if task-induced change in gamma or low gamma differed across three distinct phases of the task (encoding, maintenance and retrieval, Fig. 4A) according to whether the mouse chose the correct or incorrect response in the choice phase. Spectral power heat maps were generated and we observed an induced low gamma signal specifically during the maintenance phase and specifically within the hippocampus, which appeared to be higher when mice made the correct choice compared to incorrect choice (Fig. 4B). We next assessed and statistically compared change in low gamma power within the hippocampus in all mice (irrespective of treatment or genotype) across the 2 s of encoding, maintenance and retrieval and according to whether they selected the correct or incorrect choice. Supplementary Table 5 shows all statistical outputs for this analysis. Change in low gamma power significantly increased across the 2 s maintenance period (main effect of time, F (2.797, 727.3) = 10.24, p < 0.0001) and was significantly increased during the maintenance period if the mouse chose the correct response compared to incorrect response (main effect of response, F (1, 260) = 5.148, *p* = 0.024, Fig. 4D) but low gamma was not altered by time or response choice during encoding, or retrieval (Fig. 4C, E). Change in low gamma was unaltered by correct or incorrect response during all three phases of encoding, maintenance and retrieval in the PFC (Fig. 4F–H).

We next sought to determine if genotype or drug influence change in hippocampal low gamma during the maintenance phase given change in low gamma was increased when mice made the correct compared to incorrect selection. Here, the later 1.5–2 s of the maintenance phase was assessed as this is the time point whereby the largest difference between correct and incorrect response can be found (Fig. 4D). We ran a mixed model analysis with the fixed effects of drug, genotype and response (correct or incorrect), and drug as a repeated measure. There was no effect or interaction with response or genotype, however, there was a significant effect of drug (F (3.101, 131.0) = 5.090, *p* = 0.002). Tukey’s multiple comparisons test showed that this was driven by a significant difference between the R-ket and S-ket group (*p* = 0.026). (Fig. 4I). No other drugs had a significant effect on low gamma power during this phase. Furthermore, there was no effect of drug or genotype on low gamma power during maintenance in the PFC (Fig. 4J).

**Fig. 5:**
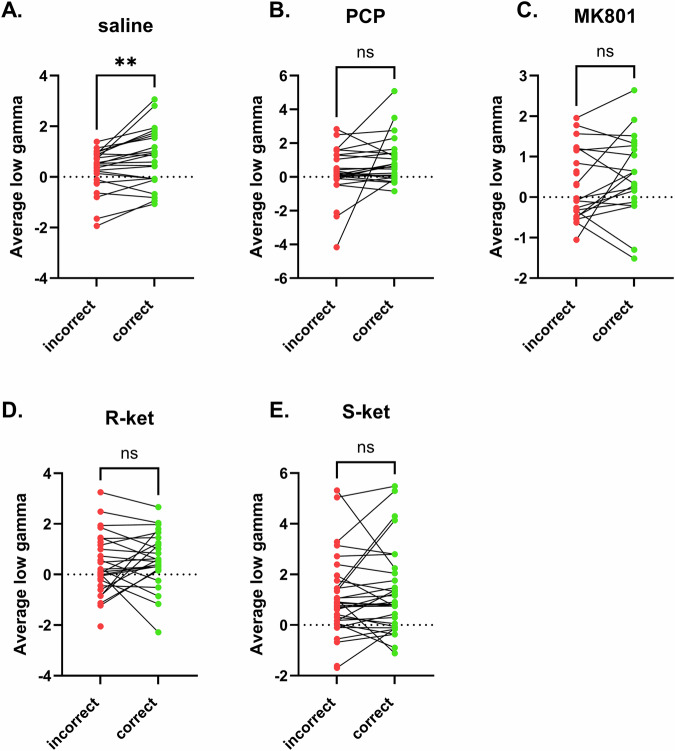
Hippocampal low gamma during the maintenance phase of incorrect versus correct trials. Low gamma significantly increases during correct trials in saline treated mice (**A**) (irrespective of genotype), but is unchanged when mice are administered PCP, (**B**); MK-801 (**C**); R-ket (**D**); or S-ket (**E**). **p < 0.01. N = 24 (saline), 24 (PCP), 19 (MK801), 25 (R-ket) and 31 (S-ket).

Given that all NMDAR antagonists disrupt working memory, we next sought to understand whether the increase in hippocampal low gamma during maintenance differed according to drug treatment. We therefore ran paired t tests for each group (saline, PCP, MK-801, R-ket and S-ket) to assess low gamma during incorrect compared to correct trials. In all saline treated mice, irrespective of genotype, low gamma increased during correct trials (Fig. 5A, *p* = 0.0028). However, when mice were administered PCP (Fig. 5B, *p* = 0.117), MK-801 (Fig. 5C, *p* = 0.213), R-ket (Fig. 5D, *p* = 0.551) or S-ket (Fig. 5E, *p* = 0.536) there was no significant change in low gamma power when comparing incorrect to correct trials. Given that the effect of PCP was dependent on genotype we included a separate analysis of WT and KO mice with PCP treatment, however there was no significant difference between incorrect and correct trials in either WT (*p* = 0.128) or KO (*p* = 0.105) PCP treated mice (Supplementary Fig. 2).

## Discussion

This study expectedly found that NMDAR antagonists consistently impair working memory; however, while there was no effect of genotype on working memory, the disrupting effect of PCP was only present in WT, but not GluN2D KO mice, suggesting this receptor subunit is required for the action of PCP in WM. Furthermore, all drugs except for R-ket increased baseline gamma and/or low gamma power in the PFC, while in the hippocampus only PCP increased baseline gamma and once again, for PCP this was genotype specific (only in WT mice, not KO mice). Finally, we identified upregulation of task-induced low gamma oscillations during the maintenance phase of the TUNL task when mice chose the correct response in the task. Task-induced low gamma only increased in saline treated mice when the correct choice was made but when mice were treated with any of the drugs this significant increase was lost.

We report no differences between the genotypes in TUNL task accuracy, indicating that the GluN2D subunit is not involved in working memory. A recent study assessing conditional KO of the GluN2D subunit from PV interneurons similarly reported no impairments in PV-GluN2D-KO mice during the Y-maze task of spatial WM when compared with WT mice [59]. However, they found impairments in short-term but not long-term memory during the Novel Object Recognition task and significant deficits in cognitive flexibility during the water T-maze test [59]. These cognitive measures were not assessed in our study. However, it suggests that while deletion of the GluN2D subunit is not sufficient to cause WM impairments, the GluN2D subunit may be important for other cognitive functions such as object recognition memory and cognitive flexibility. In this study, we show that all NMDAR antagonists PCP, MK-801, R-ket and S-ket disrupt TUNL performance. Treatment with PCP, however, leads to a decrease in overall accuracy in WT but not GluN2D-KO mice, suggesting that the WM-impairing effects of PCP are mediated by the GluN2D subunit. The drug response may be explained by the relative affinity of each drug for specific NMDAR subunits. For example, previous reports suggest that PCP is least potent for GluN2A but shows comparable potency for GluN2B-D subunits, while MK-801 is 10-fold more potent for GluN2A or B containing receptors [35, 60], and ketamine has a higher potency at GluN1/2 C as well as GluN1/2B subunit containing receptors [61, 62]. Previous reports suggest that PV interneurons are particularly affected by PCP treatment, with prenatal PCP treatment selectively reducing PV density in the PFC and hippocampus, and these changes are linked with schizophrenia-like behavioural deficits including WM impairment [63–65]. Our results suggest that PCP in disrupting WM primarily acts through GluN2D-containing NMDARs, which are mainly expressed on PV interneurons, altering the E/I balance in the brain [59]. In contrast, MK-801 impaired overall accuracy in both genotypes which suggests that WM deficits following MK-801 are not mediated by the GluN2D subunit. A previous study showed that MK-801 dose-dependently impaired TUNL accuracy in both WT mice and mice lacking the obligatory GluN1 subunit of NMDARs from PV interneurons and forebrain pyramidal cells [66]. This suggests that NMDAR hypofunction on interneurons may not be the primary mechanism underlying the WM deficits following MK-801 treatment. Alternatively, studies have linked the cholinergic and dopaminergic systems as well as changes in glial cells like astrocytes to the WM-inducing effects of MK-801 [67–69].

R-ket and S-ket did not impact accuracy in either genotype when considering effects over the entire session, however when examining accuracy over the first 12 trials alone, a significant reduction in accuracy was observed. This may reflect the shorter half-life of ketamine when compared with PCP or MK-801. The elimination half-life of ketamine is thought to be approximately 13 min in mice when administered i.p. compared with 46 min for PCP, whilst MK-801 has been shown to persist for 3 h following a single administration [70–72].

All NMDAR antagonists increased baseline gamma power in the prefrontal cortex except for R-ket, while only PCP increased gamma power in the hippocampus. This aligns with the increase in baseline gamma power reported in people with schizophrenia [73] and in previous animal model studies showing acute treatment with NMDAR antagonists increases gamma power in both the hippocampus and frontal cortex [27, 74–76]. We extend this work to show novel findings that PCP only increases baseline gamma power in the hippocampus of WT mice, and GluN2D-KO mice are protected from this increase in baseline gamma power. Furthermore, the effect of PCP was also influenced by genotype in the PFC, but in an opposite manner, whereby GluN2D-KO mice show a heightened baseline gamma power increase in response to PCP. These genotype specific effects of PCP on gamma power may be an underlying mechanism by which GluN2D- KO mice are protected from the WM deficits induced by PCP. Previously, Sapkota et al., reported that ketamine induced a much larger increase in high gamma power in WT compared with GluN2D-KO mice and suggested that the GluN2D subunit may be critical for ketamine’s effect on neural oscillations [50]. Our data showed a main effect of genotype on low gamma power in the hippocampus whereby WT mice showed higher gamma power than GluN2D KO, however, within the PFC GluN2D-KO mice seemed to show a heightened response to the drugs. A major point of difference here is that we used depth electrodes and show striking regional differences, while Sapkota et al. used electrocorticographic analysis. Our data indicate differential effects of PCP depending on genotype, and show that hippocampal changes in baseline gamma power align with working memory performance.

Task-induced changes in gamma power were evident during the maintenance phase of the task and specifically within the low gamma frequency range (30–40 Hz). Low gamma during maintenance was significantly higher when mice went on to make the correct response in the choice phase, suggesting this is an electrophysiological correlate of working memory maintenance. In addition, low gamma significantly increased during the correct trials only in saline treated mice, with no change in low gamma from incorrect to correct trials when mice were administered any of the NMDAR antagonist drugs. This further supports our suggestion that induced low gamma during the maintenance phase is an electrophysiological correlate of working memory maintenance as it is disrupted by drugs that disrupt working memory. This aligns with previous reports that people with schizophrenia show reduced task-induced gamma power specifically during the maintenance phase of WM [77]. However, the study by Haenshel et al. also reported reduced theta and gamma activity during the retrieval phase in people with schizophrenia [77] and we did not find any significant effect of NMDAR antagonists on induced gamma power during retrieval. This highlights an important difference between drug-induced models and people with schizophrenia. Maintenance in working memory is the process of actively holding information for a short period of time allowing for further processing or manipulation of the information before it dissipates. Gamma band (20–80 Hz) synchronisation is induced in humans during the delay period of the delayed-matching-to -sample task (similar to the mouse TUNL task), whereby an object representation is held in short-term memory [78]. In this study, the authors proposed that representation of visual objects through the oscillatory synchronization of a distributed neural assembly enables rehearsal of the first stimulus reperesentation in memory [78]. Our data may reflect similar rehearsal of the first stimulus through induced synchronisation of neural assemblies. In our mouse study, we see this induced activity specifically within the low gamma range (30–40 Hz) and only in the hippocampus, not the PFC, while [78] report induced gamma (20–80 Hz) in frontal and occipitotemporal electrodes suggesting species differences, as well as task variation in this induced response.

In conclusion, we report here that NMDAR antagonists disrupt WM accuracy, and characterise electrophysiological changes in the hippocampus and PFC which accompany these behavioural consequences of these drugs. We also add that PCP acts through the GluN2D subunit to exert its effects on baseline hippocampal gamma power and WM accuracy. Furthermore, the TUNL WM task induced a low gamma signal during the maintenance phase of the task and this signal was increased when mice went on to make the correct response in the choice phase, suggesting it is an important parameter in the ability of the mouse to complete the task correctly. Given the strong link between NMDAR hypofunction and schizophrenia and the burgeoning literature surrounding the specific role of the GluN2D subunit in schizophrenia [79], these data provide new and important insights into the molecular biology that may underlie working memory disruptions in schizophrenia.

## Supplementary information

Supplementary material

## Data Availability

All datasets are presented in the main manuscript or as supplementary tables or figures.
