# Supplementary material for: Effects of NMDA receptor antagonists on working memory and gamma oscillations, and the mediating role of the GluN2D subunit
Source: Neuropsychopharmacology. 2025 May 15;50(13):1938–48. doi: 10.1038/s41386-025-02129-9 (PMC12603283; doi:10.1038/s41386-025-02129-9)

**Supplementary methods**

***Electrode implant surgery***

Mice were anaesthetized with 5% isoflurane, and 125 μm stainless steel recording electrodes (Cat # E363/3/SPC, 20 mm, PlasticsOne, Bioscientific, NSW, Australia) were implanted into the mPFC (AP: +1.9, ML: -0.4, DV: -1.7) and dHPC (AP: -1.8, ML: 1.3, DV: -1.3). A ground/reference electrode (Cat # E363/120/2.4, PlasticsOne, Bioscientific, NSW, Australia) was screwed into the cerebellum and two anchor screws were inserted on either side of the frontal plate. Electrodes were then connected to a multi-channel electrode pedestal (Cat # M52-5002545, Element 14, Australia) and secured to the skull using super glue and dental cement. Mice were administered buprenorphine for pain relief at the end of the surgery and allowed to recover for 7 days with food and water available *ad libitum*.

***Electrophysiological procedures***

To record continuous LFPs during the test sessions, mice were connected via the head-mounted electrode pedestal to a custom-designed electrophysiology cable within the touchscreen chamber which was responsible for signal conditioning, multiplexing and digitising the analogue electrophysiological signal and transmitting the data to the acquisition hub where it was synchronised with behavioural data. The head stage cable was attached to a dragonfly commutator placed atop the touchscreen enclosure which prevented twisting of the cable when the mouse was performing the touchscreen task. Recordings were performed using Multi-Channel Systems Experimenter software (version # 2.8.2.18079, Harvard Biosciences Inc., USA), and sampled at 2000Hz.

***Electrophysiological analysis***

Electrophysiology analysis consisted of two measurements: baseline activity and task-induced activity. Data was extracted from the relevant periods (see below for description). All epochs were first visually inspected for artefacts, with any epochs containing appreciable artefact manually rejected from subsequent analysis. While analysing the data, we found a small number of trials with a very long response time (maximal response time: 551.4 s). These longer response times were considered to be significantly beyond the WM capacity of mice. Upon plotting the response time of all trials (Supplementary Fig 1), we found that 75% of trials were completed within 7s and thus, any trial that took greater than 7 s was excluded from further analysis.

***Measurement of baseline activity***

First, we examined the effect of the NMDAR antagonists on baseline oscillatory power in WT and GluN2D-KO mice. For this, we extracted data from a 5s window within the 12s intertrial interval (ITI) preceding each trial. All epochs were subject to spectral analysis using the multitaper method to compute the power spectral density (PSD) between 1 and 200Hz. This was achieved using the mtspectrumc function from the MATLAB Chronux plugin. The PSD estimate was then averaged across all baseline periods within each TUNL session to compute an average PSD. Ongoing power in the low gamma (30-40 Hz) and gamma (30-80 Hz) frequency band was subsequently calculated by taking the integral of all values within the appropriate frequency interval. To generate power-spectral density plots, data was log transformed for graphical representation.

***Measurement of task-induced activity***

We next measured the effects of NMDAR antagonists on TUNL task-related oscillatory activity in WT and GluN2D-KO mice. Continuous LFP data was segmented into epochs from 0-2000 ms prior to selecting the sample stimulus (i.e.: the encoding phase), 0-2000 ms immediately following selection of the sample stimulus (i.e.: maintenance phase) and 0-2000 ms prior to selection of the choice stimulus (i.e.: retrieval phase) - see Figure 4A. Epochs were also categorised according to whether the mouse made a correct or incorrect choice in that specific trial. Then a time-frequency analysis was performed whereby the data was subjected to morlet wavelet decomposition using the EEGLab newtimef function in MATLAB. This calculated event related spectral perturbations (ERSPs) at 180 linearly spaced frequencies from 5 – 50 Hz with wavelet cycles increasing from 3 to 10. ERSPs indicated a task-evoked substantial increased power within the low gamma (30-40Hz) frequency band specifically during the maintenance phase of the task, therefore power at this frequency was extracted and represented as the change in power relative to the baseline period described above. Theta and gamma activity were also extracted and analysed through each phase of the task but power did not differ between correct and incorrect trials, therefore no further drug or genotype effect analysis was applied.

**Supplementary Table 1.** TUNL training protocol.

| **Schedule** | **Criteria** |
| --- | --- |
| Habit 1 (20 min) | 2 days |
| Initial touch  (30 trials out of 60 min) | If 30 trials in 60 min  Then next stage (usually 1 day) |
| Must touch  (30 trials out of 60 min) | If 30 trials in 60 min  Then next stage (about 5-10 days) |
| Must Initiate  (30 trials out of 45 min) | If 30 trials in 45 min  Then next stage (about 2 days) |
| Punish Incorrect  (48 trials out of 30 min) | If 2 consecutive days > 80% correct  Then next stage |
| **Separation training Stage 1 (36 trials out of 45 min)** | ***All mice to pass Stage 1 before progressing cohort to Stage 2 training. If mouse has completed all Stage 1 testing before the rest of the cohort, re-test every 2 days while waiting for the rest to catch up.* |
| Exp1Stage1 S3 | If 2 consecutive days >70% correct, then next stage |
| Exp1Stage1 S2 | If 2 consecutive days >70% correct, then next stage |
| Exp1Stage1 S1 | If 2 consecutive days >70% correct, then next stage |
| **Separation training Stage 2 (48 trials out of 60 min)** | ***All mice should be trained for the same number of days* |
| Exp1Stage2 S1 (1 separation) | Daily training till group performance stabilises (>80%) |
| Exp1Stage2 S0 (0 separation) | Daily training till group performance stabilises *** stopped training after 7 days as mice showed no signs of learning/acquiring task.* |
| **Probe trials (48 trials out of 60 min)** | **Separation levels**  **(12 sessions in total)** |
| Delay 2 | 1 session per separation level (S1c, S1nc, S2, S3) in random order |
| Delay 3 | 1 session per separation level (S1c, S1nc, S2, S3) in random order |
| Delay 1 | 1 session per separation level (S1c, S1nc, S2, S3) in random order |
| **Drug Trials** **(48 trials out of 60 min)** | Schedule: S1c with 1sec delay |
| R-ketamine | 3, 10 and 30 mg/kg of R-ketamine and saline in random order |
| S-ketamine | 3, 10 and 30 mg/kg of S-ketamine and saline in random order |
| MK801 | 0.1 and 0.3 mg/kg of MK-801 and saline in random order |
| PCP | 1 and 3 mg/kg of PCP and saline in random order |
|  |  |

**Supplementary Figure 1**. Response latency (time between nose-poke to sample and choice stimulus) of mice (n=40) doing the TUNL task while tethered to the head-stage cable to allow for electrophysiological recordings.


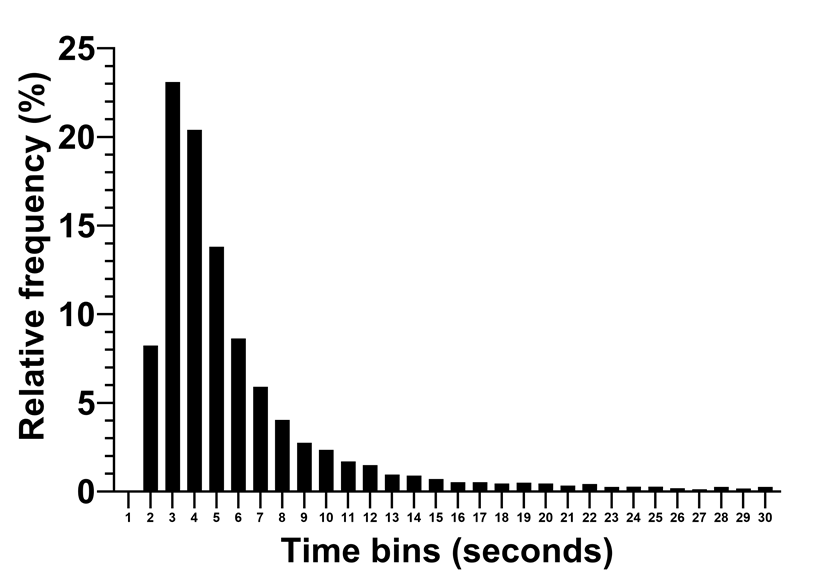


**Supplementary Table 2. The effects of different doses of NMDAR antagonists on working memory accuracy.**

| **Drug dose** | **Working memory mean % accuracy ± S.E.M.** | | | |
| --- | --- | --- | --- | --- |
|  | **Male WT (n=12)** | **Female WT (n=10)** | **Male KO (n=12)** | **Female KO (n=8)** |
| Saline | 80.38 ±2.35 | 78.54 ±2.06 | 82.98 ±1.51 | 77.60 ±2.80 |
| 1mg/kg PCP | 74.3 ±1.56 | 71.04 ±2.11 | 79.86 ± 2.38* | 81.84 ±1.55* |
| 3mg/kg PCP | 68.77 ±2.66 | 66.45 ±3.20 | 72.39 ± 2.19 | 67.96 ±2.72 |
|  |  |  |  |  |
| MK-801 | 76.73 ±2.20 | 79.79 ±2.01 | 83.68 ±1.82 | 77.08 ±3.17 |
| 0.1mg/kg MK-801 | 72.39 ±1.85 | 69.79 ±3.67 | 77.60 ±2.69 | 71.87 ±2.81 |
| 0.3mg/kg MK-801 | 57.21 ±2.59 | 63.33 ±2.56 | 61.32 ±3.16 | 65.62 ±2.52 |
|  |  |  |  |  |
| R-ket | 80.55 ±3.45 | 73.33 ±2.99 | 77.77 ±3.60 | 79.16 ±2.22 |
| 3mg/kg R-ket | 67.36 ±2.60 | 76.66 ±3.88 | 73.61 ±3.67 | 67.70 ±5.08 |
| 10mg/kg R-ket | 78.47 ±4.03 | 68.33 ±4.08 | 75 ±3.24 | 63.54 ±5.87 |
| 30mg/kg R-ket | 65.27 ±2.87 | 69.16 ±4.97 | 63.19 ±4.75 | 67.70 ±6.18 |
|  |  |  |  |  |
| S-ket | 75 ±3.24 | 75.83 ±4.20 | 75 ±3.07 | 70.83 ±5.89 |
| 3mg/kg S-ket | 70.83 ±4.64 | 76.66 ±6.47 | 75 ±3.07 | 76.04 ±2.92 |
| 10mg/kg S-ket | 61.11 ±5.26 | 72.5 ±4.48 | 74.30 ±3.31 | 77.08 ±4.91 |
| 30mg/kg S-ket | 72.91 ±3.57 | 65.83 ±4.38 | 63.19 ±3.15 | 66.66 ±1.57 |

**Supplementary Table 3. Summary statistics working memory accuracy**

| ***Working memory accuracy*** | | | | |
| --- | --- | --- | --- | --- |
| **PCP group** | | | | |
|  | **Fixed effects** | **P value** | **F (DFn, DFd)** | **Šídák's multiple comparisons test** |
| * | Drug x genotype | 0.034 | F (1, 79) = 4.673 | Saline WT v KO, P=0.79  ***PCP WT v KO, P=0.001***  ***WT saline v PCP, P=0.003***  KO saline v PCP, P=0.992 |
| * | Drug | 0.023 | F (1, 79) = 5.395 |  |
| ** | Genotype | 0.0034 | F (1, 79) = 9.126 |  |
| **MK-801** | | | | |
|  | Drug x genotype | 0.972 | F (1, 40) = 0.001140 |  |
| *** | Drug | <0.0001 | F (1, 40) = 87.16 |  |
|  | Genotype | 0.098 | F (1, 40) = 2.868 |  |
| **R-ket all 50** | | | | |
|  | Drug x genotype | 0.384 | F (1, 40) = 0.7743 |  |
|  | Drug | 0.395 | F (1, 40) = 0.7393 |  |
|  | Genotype | 0.25 | F (1, 40) = 1.359 |  |
| **S-ket all 50** | | | | |
|  | Drug x genotype | 0.522 | F (1, 40) = 0.4161 |  |
|  | Drug | 0.455 | F (1, 40) = 0.5680 |  |
|  | Genotype | 0.512 | F (1, 40) = 0.4383 |  |
| **R-ket first 12** | | | | |
|  | Drug x genotype | 0.608 | F (1, 40) = 0.2662 |  |
| *** | Drug | 0.0003 | F (1, 40) = 15.32 |  |
|  | Genotype | 0.851 | F (1, 40) = 0.03571 |  |
| **S-ket first 12** | | | | |
|  | Drug x genotype | 0.570 | F (1, 40) = 0.3270 |  |
| ** | Drug | 0.010 | F (1, 40) = 7.235 |  |
|  | Genotype | 0.167 | F (1, 40) = 1.979 |  |

**Supplementary Table 4. Summary statistics baseline gamma power**

| ***Hippocampus*** | | | | |
| --- | --- | --- | --- | --- |
| **Gamma power** | | | | |
|  | **Fixed effects** | **P value** | **F (DFn, DFd)** | **Šídák's multiple comparisons test** |
| * | Drug x genotype | 0.013 | F (4, 99) = 3.336 | ***WT saline v PCP, P=0.046***  KO saline v PCP, P=0.99 |
| ** | Drug | 0.002 | F (3.309, 81.90) = 5.012 | ***Saline v PCP, P=0.049***  Saline v MK-801, P=0.117  Saline v R-ket, P=0.99  Saline v S-ket, P=0.26 |
|  | Genotype | 0.241 | F (1, 36) = 1.419 |  |
| **Low gamma power** | | | | |
|  | Drug x genotype | 0.105 | F (4, 94) = 1.972 |  |
|  | Drug | 0.292 | F (2.678, 62.92) = 1.268 |  |
| * | Genotype | 0.011 | F (1, 36) = 7.138 |  |
| **PFC** | | | | |
| **Gamma power** | | | | |
|  | **Fixed effects** | **P value** | **F (DFn, DFd)** | **Šídák's multiple comparisons test** |
| * | Drug x genotype | 0.027 | F (4, 123) = 2.847 | ***WT saline v PCP, P=0.005***  KO saline v PCP, P=0.918  ***WT saline v MK-801, P=0.002***  KO saline v MK-801, P=0.292  ***WT saline v R-ket, P=0.517***  KO saline v R-ket, P=0.920  ***WT saline v S-ket, P=0.07***  KO saline v S-ket, P=0.059 |
| *** | Drug | <0.0001 | F (2.752, 84.62) = 15.94 | ***Saline v PCP, P=0.018***  ***Saline v MK-801, P=0.002***  Saline v R-ket, P=0.208  ***Saline v S-ket, P=0.001*** |
| ****** | Genotype | 0.0001 | F (1, 123) = 15.91 |  |
| Low gamma power | | | | |
|  | Drug x genotype | 0.070 | F (4, 87) = 2.243 |  |
| *** | Drug | <0.0001 | F (2.884, 62.73) = 14.03 | ***Saline v PCP, P=0.020***  ***Saline v MK-801, P=0.002***  Saline v R-ket, P=0.753  **Saline v S-ket, P=0.010** |
|  | Genotype | 0.176 | F (1, 36) = 1.899 |  |

**Supplementary Table 5. Summary statistics task-induced low gamma according to correct or incorrect response**

| ***Hippocampus*** | | | | |
| --- | --- | --- | --- | --- |
| **Task-induced low gamma during encoding** | | | | |
|  | **Fixed effects** | **P value** | **F (DFn, DFd)** | **Tukey’s multiple comparisons test** |
|  | Time | 0.129 | F (2.787, 752.6) = 1.924 |  |
|  | Response | 0.504 | F (1, 276) = 0.4471 |  |
|  | Time x response | 0.3466 | F (3, 810) = 1.104 |  |
| **Task-induced low gamma during maintenance** | | | | |
| *** | Time | <0.0001 | F (2.797, 727.3) = 10.24 | ***0-0.5 v 0.5-1, P=0.048***  ***0-0.5 v 1-1.5, P<0.0001***  ***0-0.5 V 1.5-2, P=0.0007***  ***0.5-1 v1-1.5, P=0.0182***  0.5-1 v 1.5-2, P=0.3347  1-1.5 v 1.5-2, P=0.8880 |
| * | Response | 0.0241 | F (1, 260) = 5.148 |  |
|  | Time x response | 0.5833 | F (3, 780) = 0.6497 |  |
| **Task-induced low gamma during retrieval** | | | | |
|  | Time | 0.2123 | F (2.910, 803.1) = 1.507 |  |
|  | Response | 0.5788 | F (1, 276) = 0.3089 |  |
|  | Time x response | 0.9323 | F (3, 828) = 0.1459 |  |
| **PFC** | | | | |
| **Task-induced low gamma during encoding** | | | | |
|  | **Fixed effects** | **P value** | **F (DFn, DFd)** | **Tukey's multiple comparisons test** |
| *** | Time | <0.0001 | F (2.668, 709.8) = 12.29 | 0-0.5 v 0.5-1, P=0.5502  0-0.5 v 1-1.5, P=0.1964  **0*-0.5 V 1.5-2, P=0.0007***  ***0.5-1 v1-1.5, P=0.0018***  ***0.5-1 v 1.5-2, P<0.0001***  1-1.5 v 1.5-2, P=0.0517 |
|  | Response | 0.2346 | F (1, 269) = 1.419 |  |
|  | Time x response | 0.2416 | F (3, 798) = 1.400 |  |
| **Task-induced low gamma during maintenance** | | | | |
|  | Time | 0.0590 | F (2.697, 719.2) = 2.578 |  |
|  | Response | 0.9838 | F (1, 267) = 0.0004152 |  |
|  | Time x response | 0.2584 | F (3, 800) = 1.346 |  |
| **Task-induced low gamma during retrieval** | | | | |
| ***** | Time | 0.0079 | F (2.624, 723.3) = 4.250 | 0-0.5 v 0.5-1, P=0.9165  0-0.5 v 1-1.5, P=0.9981  0-0.5 V 1.5-2, P=0.0423  0.5-1 v1-1.5, P=0.7561  0.5-1 v 1.5-2, P=0.0122  1-1.5 v 1.5-2, P=0.0980 |
|  | Response | 0.3526 | F (1, 276) = 0.8668 |  |
|  | Time x response | 0.8276 | F (3, 827) = 0.3108 |  |

**Supplementary Figure 2.** Induced low gamma during maintenance does not significantly differ in incorrect versus correct trials in WT mice treated with PCP (A), and KO mice treated with PCP (B).


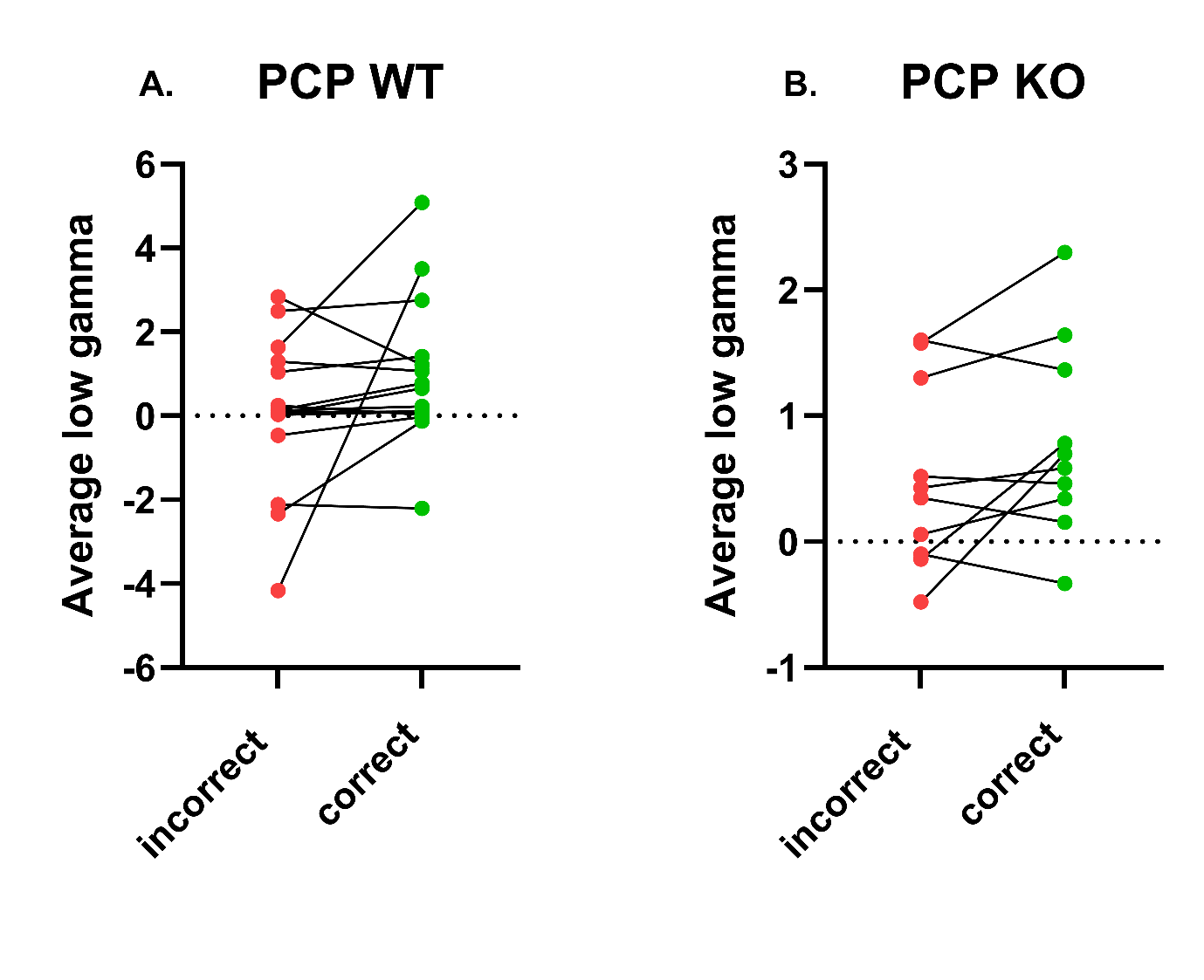

Supplement: Supplementary file 1 — Supplementary material [file 41386_2025_2129_MOESM1_ESM.docx]
